# Cutaneous Findings as a Harbinger of Disseminated Fungal Infection

**DOI:** 10.7759/cureus.73944

**Published:** 2024-11-18

**Authors:** Molly Antonson, Robert Borucki, Corey J Georgesen

**Affiliations:** 1 Department of Dermatology, University of Nebraska Medical Center, Omaha, USA

**Keywords:** disseminated histoplasmosis, fungal infection, h capsulatum, histoplasma, primary cutaneous histoplasmosis, skin biopsy

## Abstract

*Histoplasma capsulatum* is a dimorphic fungus that causes pulmonary, disseminated, or, rarely, primary cutaneous disease. Primary cutaneous histoplasmosis presents with non-specific skin lesions, which can lead to poor patient outcomes due to diagnostic challenges and delays in diagnosis. A 62-year-old male on immunosuppressive medications for lupus nephritis presented to the emergency department with 24 hours of altered mental status. He had a three-month history of non-healing, ulcerated plaques with hemorrhagic discharge on the left side of his face. Although multiple urine antigen tests were negative, the patient was ultimately diagnosed with disseminated histoplasmosis and histoplasma meningitis based on skin biopsy and lumbar puncture findings. Given the longstanding nature of the patient’s skin lesions prior to systemic symptoms, this likely represents a rare case of disseminated histoplasmosis secondary to primary cutaneous infection, highlighting the importance of including *H. capsulatum* in the differential diagnosis when an immunocompromised patient presents with nonspecific skin lesions.

## Introduction

Histoplasmosis is a systemic infection caused by the dimorphic fungus *Histoplasma capsulatum*, most commonly found in contaminated soil in the Ohio and Mississippi River Valley and in tropical countries [1,2]. Clinical variants of histoplasmosis include pulmonary (most common), disseminated, and primary cutaneous histoplasmosis [3]. Primary cutaneous histoplasmosis, acquired by direct inoculation of *H. capsulatum* during injury to the skin, is extremely rare and may present as erythematous papules or plaques with or without crust, nodules, wart-like plaques, erosions, or ulcers [4]. Early diagnosis of primary cutaneous histoplasmosis is crucial to prevent dissemination and poor patient outcomes, as the disease can be fatal. Here, we report a case of a 62-year-old male with primary cutaneous histoplasmosis of the face that disseminated to cause histoplasma meningitis, emphasizing the importance of biopsy for atypical or nonspecific skin lesions to aid in the diagnosis of systemic disease, especially for immunosuppressed patients.

This research was presented as an abstract at the Graduate Medical Education Research Symposium in March 2023 at the University of Nebraska Medical Center and at the Medical Student Research Showcase in August 2023 at the University of Nebraska Medical Center.

## Case presentation

**Figure 1:**
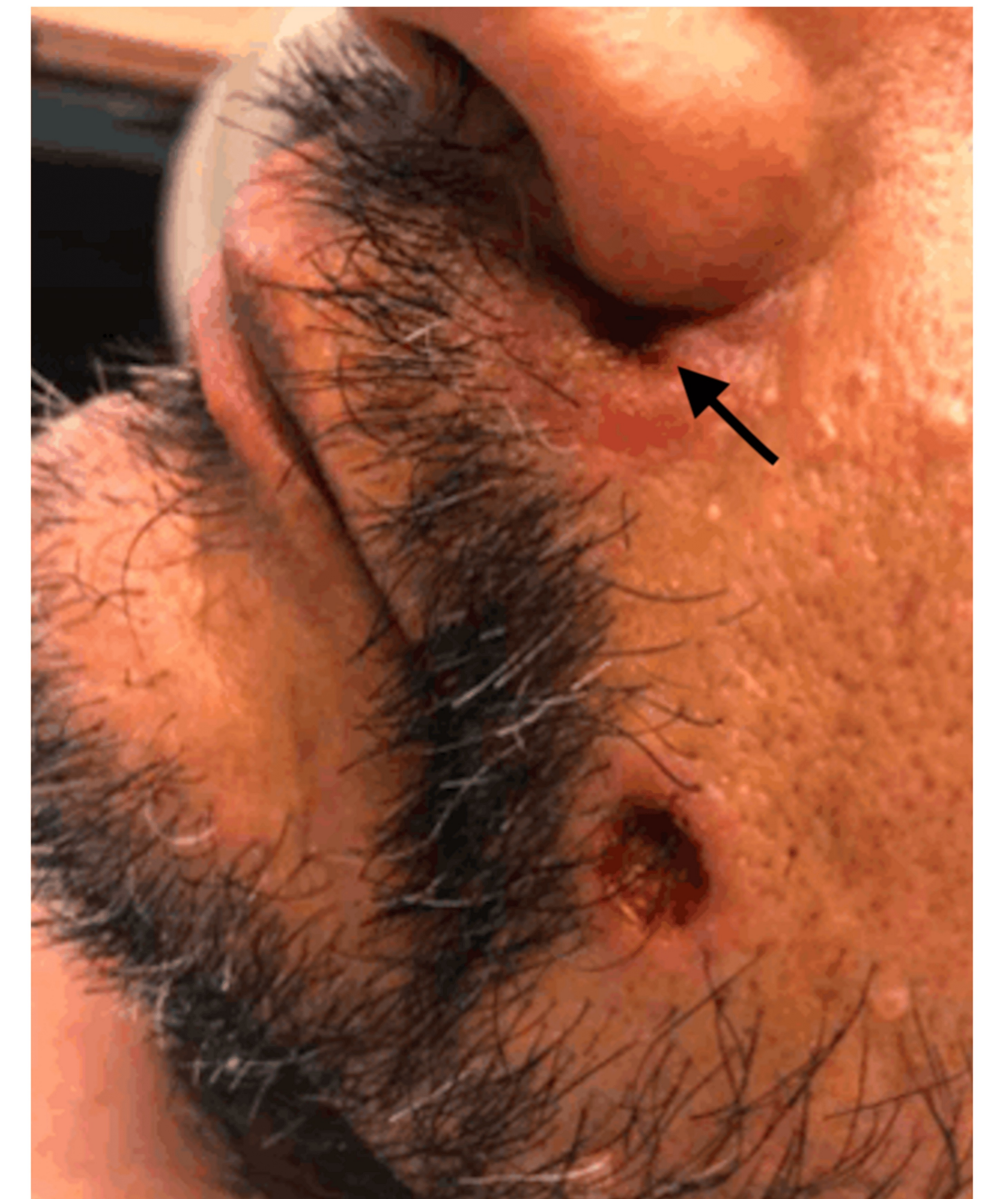
Left alar facial groove (with arrow) and left lower cheek at initial presentation

A 62-year-old Puerto Rican male with end-stage renal disease secondary to lupus nephritis on mycophenolate mofetil presented to the emergency department with 24 hours of altered mental status. A physical exam demonstrated a pink, ulcerated plaque with rolled borders and a central depression with hemorrhagic crust in the left alar facial groove and a similar lesion on the left lower cheek, both of which had been present for approximately three months (Figure 1). The patient reported hemorrhagic discharge and enlargement of both lesions over the past month, along with daily fevers. His social history was notable for frequent travel to the Caribbean.

**Figure 2:**
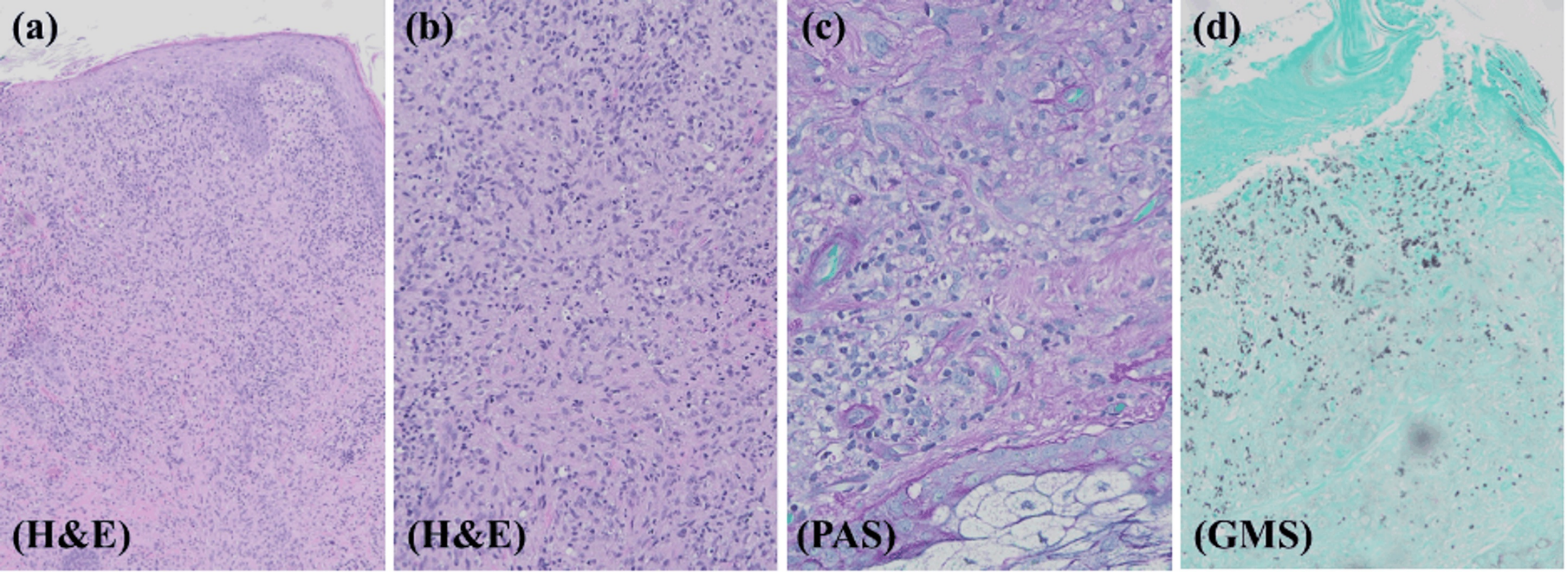
Histopathology of the lesion on the left cheek demonstrating granulomatous inflammation with multinucleated giant cells containing intracellular organisms with (a) hematoxylin and eosin stain, 10x magnification, (b) hematoxylin and eosin stain, 20x magnification. (c) Periodic acid-Schiff (PAS), 40x magnification, and (d) Grocott Methenamine Silver (GMS), 20x magnification, stains were positive for fungal yeast organisms with capsules.

Upon dermatology consultation, the differential diagnosis included extranodal natural killer T-cell lymphoma, deep fungal infection (cryptococcus, histoplasmosis, or coccidiomycosis), atypical mycobacterial infection, and leishmaniasis. Based on this differential and concern for an infectious or neoplastic process, a skin biopsy was performed. A 4 mm punch biopsy of the left cheek revealed granulomatous inflammation with multinucleated giant cells containing intracellular organisms. Culture results showed non-sporulating mold. Periodic acid-Schiff (PAS) and Grocott Methenamine Silver (GMS) stains were positive for fungal yeast organisms with capsules, consistent with histoplasma (Figure 2).

A head computed tomography scan was normal, and rheumatologic markers, including C3, C4, and CRP, were within normal limits. A negative double-stranded deoxyribonucleic acid (dsDNA) test, along with a recent history of well-controlled lupus symptoms, placed an acute lupus flare low on the differential. Given his altered mental status and fevers, the patient underwent a lumbar puncture to assess for central nervous system involvement. The lumbar puncture was significant for an elevated white count, low glucose, and high protein (Table 1). Histoplasma antigen was positive in both the cerebrospinal fluid (CSF) and serum. Despite multiple samples, the urine histoplasma antigen was negative. The patient was diagnosed with disseminated histoplasmosis and histoplasma meningitis. He received treatment with 5 mg/kg intravenous liposomal amphotericin B daily for six weeks. At a follow-up visit with infectious disease after six weeks of therapy, the patient showed significant clinical improvement. He was treated with oral itraconazole 200 mg twice daily for one year.

**Table 1:** Important laboratory values during hospitalization WBC: white blood cells, CSF: cerebrospinal fluid, mg/dL: milligrams per deciliter, ng/mL: nanograms per milliliter.

Laboratory Study	Laboratory Finding	Reference Range
WBC, CSF	74	0–8 cells/mm
Glucose, CSF	25	40–70 mg/dL
Protein, CSF	184	15–45 mg/dL
Histoplasma antigen, CSF	3.87	0 ng/mL
Histoplasma antigen, serum	2.0	<0.3 ng/mL
Histoplasma antigen, urine	<0.3	<0.3 ng/mL

## Discussion

Given the longstanding nature of the patient’s skin lesions before systemic symptoms, this case likely demonstrates disseminated histoplasmosis secondary to cutaneous infection. Primary cutaneous histoplasmosis leading to disseminated disease is extremely rare, with Saheki et al. reporting only 18 cases in the literature as of 2008 [5,6]. Disseminated disease likely occurs in immunocompromised patients due to inoculation of histoplasma in the skin followed by spread to the lymph nodes [7]. Because immunocompromised patients have a decreased immune response to opportunistic infections like histoplasma, disseminated disease is more commonly reported in immunocompromised patients compared to immunocompetent individuals [7]. The typically benign and self-limited course of primary cutaneous histoplasmosis in patients who are not immunocompromised likely explains why the disease is rarely reported in immunocompetent patients [6].

The clinical manifestations of cutaneous histoplasmosis vary greatly, making the diagnosis difficult when there is a lack of clinical suspicion. Interestingly, skin manifestations of histoplasmosis are more commonly reported in patients living in Latin America and Africa [8,9]. Our patient’s history of frequent travel to the Caribbean emphasizes the importance of obtaining a thorough social history to help guide the workup and diagnosis. Other cases of primary cutaneous histoplasmosis reported in the literature typically involve a history of an inciting event, such as exposure to bats or travel to an endemic country [6]. Although our patient could not recall specific trauma to the skin while traveling in the Caribbean, the ulcerations on his face appeared weeks prior to systemic symptoms. This aligns with other cases presented in the literature, as reports of primary cutaneous histoplasmosis leading to disseminated disease often describe a history of days to weeks of skin lesions before the development of lymphadenopathy or fever [6]. Importantly, the ulcerated plaques observed during the patient’s physical exam suggested the possibility of a diagnosis of histoplasmosis. Histoplasma antigen is not routinely included on a meningitis panel and may not have been considered if the skin findings had been overlooked when developing the differential diagnosis.

The diagnosis of histoplasmosis can be made through histopathology (Giemsa-stained smears or hematoxylin and eosin-stained tissue sections), culture, serology, or antigen detection [2,5]. GMS and PAS stains are especially useful for diagnosing fungal infections such as histoplasma, as the GMS stain specifically highlights fungal cell walls (which appear brown to black against the pale green background of the counterstain) and the PAS stain targets polysaccharides in fungal cell walls (fungi appear red to purple against a green counterstained background) [10]. Centers for Disease Control and Prevention (CDC) guidelines state that urine and/or serum antigen detection is the most sensitive and specific tool for diagnosing histoplasmosis, and positive antigenuria has typically been correlated with increased disease severity [1]. However, as demonstrated in this case, the sensitivities of histoplasma urine antigen tests are highly variable and differ based on the subtype of histoplasmosis and the method of immunosuppression [11]. A study by Torres-González et al. found that the sensitivity and specificity of histoplasma urine antigen tests are 67.1% (95% confidence interval, 56-76.7%) and 97.5% (95% confidence interval, 94.3-99.1%), respectively [12]. Based on these data, a positive urine antigen test is highly suggestive of the diagnosis, but a negative test result does not rule out histoplasmosis. Therefore, alternative tests such as skin biopsy with polymerase chain reaction (PCR) testing and culture-based approaches should be utilized to rule out false negatives [4,11,12]. Early biopsy and histopathologic analysis would likely have allowed for earlier treatment and prevented disseminated disease and increased morbidity in this patient.

The treatment of histoplasmosis varies based on organ involvement and severity. For patients with disseminated disease involving the central nervous system, such as our patient, liposomal amphotericin B (5.0 mg/kg daily administered over four to six weeks) followed by itraconazole (200 mg two or three times daily) for at least one year and until the resolution of CSF abnormalities is recommended [13]. Despite the risk of renal injury associated with amphotericin B treatment, the poor prognosis of severe, untreated histoplasmosis justifies its use [13].

## Conclusions

Due to the rarity of primary cutaneous histoplasmosis and its remarkably heterogeneous presentation, diagnosis is challenging. This case highlights the importance of including *H. capsulatum* in the differential diagnosis when a patient presents with nonspecific skin lesions, especially if they are immunocompromised. A low threshold for biopsy should be maintained when there is concern for histoplasma, as biopsy provides an expedited method for diagnosis and can allow for earlier treatment and improved patient outcomes.
